# Epileptic Mania Following the Suspension of Bromide Potash

**Published:** 1873-07

**Authors:** G. D. Hodge

**Affiliations:** Holly Springs, Arkansas


### EPILEPTIC MANIA FOLLOWING THE SUSPENSION OF BROMIDE POTASH.

A. M. S., aged twenty-three years, formerly a stout youth, has been the subject of confirmed epilepsy for several years; has been taking brom. pot., in the form of Brown-Sequard's preparation, constantly for two years or more, with the effect of prolonging the intervals and mitigating the attacks. His kind parents at length concluded to give him a little respite by suspending his medicine for a short time. On doing this, in less than twenty-four hours he was seized with violent mania. After a thorough cathartic, we resorted to chloral, morphia, etc., which would procure sleep, and thus nightly quietude only. We at length decided that this mental derangement might be due to the sudden withdrawal of the anti-congestive and other sedative effects well known to be exerted by the bromides on the brain. On this conviction, we ordered this medicine to be resumed. The first dose (taken at night) within six hours rendered the patient more tranquil, and after the morning potion, all mental disturbance vanished, and has continued so with the taking of the medicine. Were we correct in our conclusion?

G. D. Hodge, M.D.

Holly Springs, Arkansas, May 26, 1873.

